# Knowledge, Attitude, and Practice of the Lebanese University Medical Students and Junior Doctors on Basic Life Support Practices

**DOI:** 10.5334/gh.1368

**Published:** 2024-11-13

**Authors:** Hadi El Assaad, Bahaa Osman, Mohamad Omar Honeine, Pierre Abi-Hanna, Mirna N. Chahine

**Affiliations:** 1 Faculty of Medical Sciences, Lebanese University, Hadath, Lebanon; 2 Leipzig University Hospital, Department of Orthopedics, Trauma Surgery and Plastic Surgery, Leipzig, Germany; 3 Hotel Dieu de France Hospital, Saint Joseph University, Department of Digestive and Endocrine Surgery, Beirut, Lebanon; 4 Basic Sciences Department, Faculty of Medical Sciences, Lebanese University, Hadath, Lebanon; 5 Foundation-Medical Research Institutes (F-MRI®), Beirut, Lebanon

**Keywords:** BLS, Medical students, Knowledge, Attitudes, Practice

## Abstract

**Background:**

Basic life support (BLS) is the recognition of sudden cardiac arrest and activation of emergency response system, followed by cardiopulmonary resuscitation (CPR), and rapid defibrillation.

**Aim:**

Our study aimed to determine the level of awareness of the Lebanese University medical students and trainees on BLS, by assessing the association between knowledge, attitude, and practice on BLS, and between the demographic variables and KAP scores.

**Methods:**

This was a cross-sectional study including 330 medical students enrolled at Lebanese University, from year four of general medicine till year five of residency. An online survey was used to collect data about demographic characteristics, knowledge (K), attitudes (A), and practice (P) about BLS. Data was analyzed using SPSS version 25.

**Results:**

Participants were 52.7% females, 47.3% males, and their mean age was 24 ± 2 years. Of the 330 participants, 38.8% received formal training regarding BLS. Medical students had low knowledge (90%), moderate to good attitudes (71.5%), and low practice (93%) regarding BLS. Multiple linear regression showed that knowledge was positively associated with age (p = 0.001), knowledge and information regarding BLS (p = 0.016), and any formal training/workshop regarding BLS (p = 0.021). Attitude was positively associated with academic year (p = 0.002) and knowledge (p = 0.003). Practice was positively associated with age (p < 0.001) and knowledge (p < 0.001).

**Conclusion:**

Most Lebanese University medical students showed low knowledge, moderate to good attitudes, and low practice regarding BLS. We recommend that CPR/BLS should be a core competency across all health care professional programs.

## Introduction

Cardiac arrest is thought to account for 15–20% of total deaths (1,2). It is an important cause of cardiovascular morbidity and mortality in both developed and developing countries. Data from previous studies suggest that more than three million sudden cardiac deaths happen worldwide each year and survival rate is lower than 8% (3,4,5,6).

Basic life support (BLS) is defined as a variety of noninvasive emergency procedures performed to assist in the immediate survival of a patient, including cardiopulmonary resuscitation (CPR), hemorrhage control, stabilization of fractures, spinal immobilization, and basic first aid. Implementing these procedures early would be lifesaving in many cases (7). Cardiopulmonary resuscitation was invented in 1960 and became a simple yet efficient way to sustain lives in the early moments following cardiac or respiratory arrest (8). In other words, BLS is the recognition of sudden cardiac arrest and activation of the emergency response system, followed by resuscitation, and rapid defibrillation (9).

A study conducted in Lebanon in 2013 showed that the survival rate to hospital discharge for OHCA (Out-of-Hospital Cardiac Arrest) victims was around 5% in Beirut, Lebanon, while those who survived had lesser than 50% chance for good neurological outcomes (10). These results suggest that it is important to work on improving BLS practices among healthcare providers, especially medical students and junior doctors in order to increase the survival rate.

The Faculty of Medical Sciences in the Lebanese University is one of the largest and most reputable faculties in the country and is affiliated with many hospitals and medical centers (>15) nationwide. There are no published studies assessing the knowledge, attitude, and performance among LU medical students and trainees when it comes to BLS. However, the medical students and residents in the affiliated medical centers are usually the first contact persons in case of a cardio-respiratory arrest in emergency department, and the ones usually responsible of offering or initiating BLS. This justifies the need to evaluate the behavior of medical students at LU.

The knowledge of the Lebanese University medical students and junior doctors refers to their understanding concerning BLS practices. Attitude refers to their readiness for BLS practices. Practice refers to the ways in which they demonstrate their knowledge and attitude through their actions. The knowledge, attitude, and practice of Lebanese University medical trainees concerning BLS were not previously assessed at the university and its affiliated hospitals.

This is a cross-sectional study conducted from May 5th until August 23rd, 2021, using an electronic survey (Google Form) among Lebanese University medical students, interns, and residents.

- Demographic characteristics: age, gender, educational level, residence
- Information and sources of information
- Knowledge: 13 items to assess the participants’ knowledge concerning the BLS.

The *knowledge* of the Lebanese University medical students and junior doctors refers to their understanding concerning the BLS practices. *Attitude* refers to their readiness towards the BLS practices. *Practice* refers to the ways in which they demonstrate their knowledge and attitude through their actions. The knowledge, attitude, and practice of Lebanese University medical trainees concerning BLS was not previously assessed in the university and its affiliated hospitals. Such study is therefore important in order to determine the awareness of the Lebanese University future and junior doctors and formulate targeted interventions and educational campaigns. Studies in Egypt (11) and Pakistan (12) have reflected a low level of knowledge due to deficits in training among medical students in regards to BLS. Such results ensure the necessity of an assessment of BLS knowledge, attitude, and practice among Lebanese University medical student, especially with the lack of such studies in Lebanon.

## Methods

### Study design and population

This was a cross-sectional study conducted from May 5^th^ till August 23^rd^, 2021 using an electronic survey (Google form) among Lebanese University medical students, interns, and residents.

### Inclusion criteria

Medical students enrolled in Lebanese University Faculty of Medical Sciences, from year four of general medicine (first year of clinical studies) till year five of medical residency (R5) were targeted.

### Exclusion criteria

The subjects excluded were students and residents not willing to participate, and those below 18 years of age.

### Sample size

Medical students, interns, and Residents at the Faculty of medical sciences, Lebanese University, represented 850 in total. Based on Slovin’s formula confidence level of 95%, an alpha level of 0.05 and N = 850 participants, were used (n = N/(1 + N e2), therefore, a minimum of 300 participants was required to fill out the questionnaire in order to be representative of the Lebanese university medical students, interns, and residents.

### Procedures of data collection measurements

Data was collected online by using ‘Google Forms.’ The link to the ‘Google Forms’ survey was sent to students and junior doctors through ‘Whatsapp.’ The questionnaire required no more than seven to eight minutes to fill out and was available in English (since all of our students between Year four of medical studies and year five of residency are proficient in English language)

An online survey extracted from Yunus, et al. (13) was used for the evaluation of Knowledge, attitude, and practice towards BLS, and a demographic section was added. The data collection form included the following sections (Supplementary file):

– Demographic characteristics: Age, gender, educational level, residence
– Information and source of information
– Knowledge: 13 items to assess the participants’ knowledge concerning the BLS. Knowledge questions included definition of BLS, emergency medical service (EMS) and CPR, BLS best practices in adults and infants, actions and BLS steps and activities. The score was set over 13. The participants with higher score had 13 over 13. In the knowledge section, every correct answer was granted 1 point and each wrong answer a 0.
– Attitude: Six items to assess the participants’ attitude concerning the BLS. Attitude items included questions about the importance of BLS, voluntary participation in BLS, and attitude to do CPR. In the attitude section, a 5-point Likert scale was adopted in which: 1 ‘strongly disagree,’ 2 ‘disagree,’ 3 ‘neutral,’ 4 ‘agree,’ 5 ‘strongly agree,’ and 0 ‘don’t know.’ The score was over 30. The participants with higher performance had 30 over 30.
– Practice: 10 items to assess the participants’ practice concerning the BLS. Practice items included questions about the importance of BLS, adopting best practices for CPR and BLS in adults and infants. In the practice section, every correct answer was granted 1 point and each wrong answer a 0. The score was over 10. The participants with higher performance had 10 over 10.
– Computed scores were graded into categories and subcategories from ‘Limited’ (Poor, Fair) to ‘Adequate’ (Good, Excellent) levels of KAP, according to the Median of the scores, and a ‘modified form’ of the widely adopted Bloom’s cutoff points (14,15) (Table 1).

**Table 1:** Grading of Knowledge (K), Attitude (A), and Practice (P) scores about BLS into Categories ‘Limited and Adequate’ and Sub-Categories ‘Poor, Fair, Good, and Excellent’.


CATEGORIES	SUB-CATEGORIES	KNOWLEDGE		ATTITUDE		PRACTICE	
		
		/13	%	/30	%	/10	%

**LIMITED**	POOR	≤9	≤69.23	≤21	≤70	≤6	≤70.0

	FAIR	10–11	[67.92–84.61]	(22–24)	[73.33–80.0]	7	80.0

**ADEQUATE**	GOOD	12	92.3	(25–27)	[83.33–90.0]	9	90.0

	EXCELLENT	13	100	(28–30)	[93.33–100]	9	100


### Data analysis

All statistical analysis was performed using IBM SPSS version 25.

The statistical analysis of this research included descriptive statistics and graphs for parameters of interest, as well as statistical testing of the primary and secondary variables.

The target variables were the knowledge, attitude, and the practice. Descriptive analysis of qualitative variables comprised the frequency and percentage of each category. Quantitative variables were summarized in tables using descriptive statistics (analyzed number n, mean, standard deviation, minimum, maximum, and two-sided 95% Confidence Interval).

Bivariate analysis was conducted in order to test the correlation between the knowledge, attitude, and practice. In addition, bivariate analysis was enrolled in order to test factors affecting each of three variables (knowledge, attitude, and practice).

The test used in the bivariate settings were Pearson’s Chi-square test, Student t-test, and ANOVA test. All statistical tests will be two-sided, and the significance level will be set at 5%.

Multivariate analysis was enrolled to predict the factors affecting the KAP scores.

## Results

### Representation of the population

The population of this study included 330 medical students, interns, and residents.

### Demographic characteristics

Medical students were distributed between 52.7% of females and 47.3% of males. Mean of participants was 24 ± 2 years with a minimum of 20 years and a maximum of 31 years (Table 2).

**Table 2:** Descriptive statistics related to demographic and academic characteristics of medical students (N = 330).


		FREQUENCY	PERCENT

Gender	Male	156	47.3

	Female	174	52.7

Age	Mean (SD)	24.01 (1.94)	

	Minimum and Maximum	20–31	

Year of medical training	4th year medical student	47	14.2

	5th year medical student	82	24.8

	6th year medical student	75	22.7

	7th year medical student	57	17.3

	1st year of residency	26	7.9

	2nd year of residency	16	4.8

	3rd year of residency	12	3.6

	4th year of residency	9	2.7

	5th year of residency	6	1.8

Year of medical training	Resident	69	20.9

	Interns	132	40.0

	Medical Student	129	39.1


Out of 330 participants, 20.9% were residents (first year to fifth year of residency), 40% were interns (sixth year and seventh year medical student), and 39.1% were medical students (fourth year and fifth year medical student) (Table 2).

### Information about BLS

Out of 330 participants, 83.3% received knowledge and information regarding BLS.

Information about BLS was obtained via university courses (53.6%), hospital workshops/sessions (35.8%), internet courses (39.1%), red cross training (24.8%), pamphlets (15.2%). Out of 330 medical students, only 38.8% received any formal (certified) training/attended any workshop regarding BLS, and among them 45.3% had the training in the last two years whereas 54.7% had the training over two years ago.

### KAP scores of BLS

Findings showed that medical students, interns and residents had poor knowledge, good attitudes, and poor practice about BLS. Out of 330 medical students, 90% had poor knowledge level and mean knowledge score was 6.14 ± 2.2 over 13 (47.23%) with a minimum of 2 over 13 and a maximum of 12 over 13. Among medical students, 34.2% had good attitudes, 37.3% had excellent attitudes, and mean attitudes score was 26.01 ± 3.3 over 30 (86.70%) with a minimum of 10 over 30 and a maximum of 30 over 30. Out of 330 medical students, 93% had poor practice level and mean practice score was 4.76 ± 1.8 over 10 (47.60%) with a minimum of 0 over 10 and a maximum of 10 over 10 (Table 3).

**Table 3:** Representation of KAP scores of BLS.


		FREQUENCY	PERCENT

Knowledge	Poor	297	90.0

	Fair	28	8.5

	Good	5	1.5

	Excellent	0	0.0

	Mean (SD)	6.14 (2.25) over 13	

	Median [IQR]	6.0 [4.0–8.0]	

	Min–Max	2.0–12.0	

Attitudes	Poor	28	8.5

	Fair	66	20.0

	Good	113	34.2

	Excellent	123	37.3

	Mean (SD)	26.01 (3.34) over 30	

	Median [IQR]	27.0 [24.0–28.0]	

	Min–Max	10.0–30.0	

Practices	Poor	307	93.0

	Fair	12	3.6

	Good	7	2.1

	Excellent	4	1.2

	Mean (SD)	4.76 (1.78) over 10	

	Median [IQR]	5.0 [4.0–6.0]	

	Min–Max	0.0–10.0	


SD (Standard Deviation), IQR (Interquartile Range).

**Figure 1:**
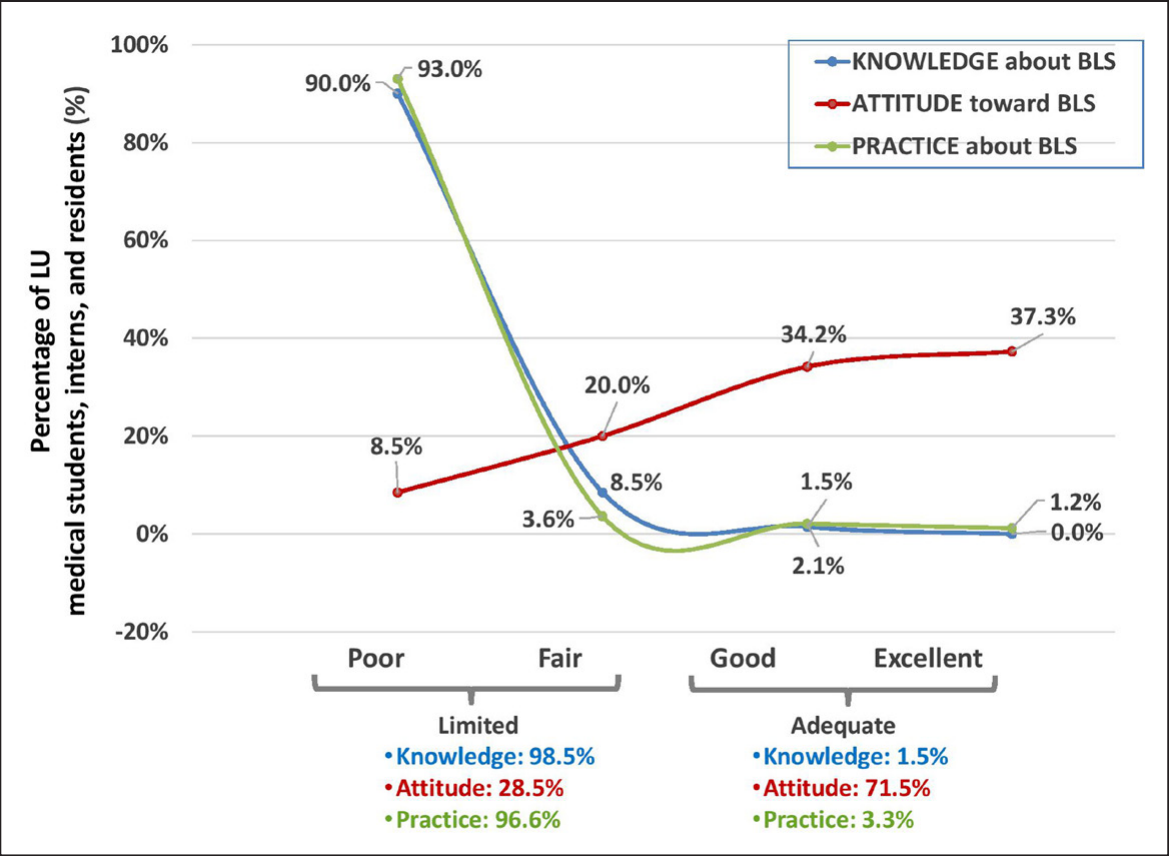
KAP towards BLS. Percentage (%) of LU medical students, interns, and residents with KAP scores represented in categories (Limited/Adequate) and sub-categories (Poor/Fair/Good/Excellent).

Only 1.5% and 3.3% of medical trainees demonstrated an ‘Adequate’ Knowledge and Practice levels about BLS, respectively. However, 71.5% of medical trainees showed an ‘Adequate’ Attitude level toward BLS (Figure 1).

Poor knowledge rate was 90.4% in males, 89.7% in females, 97.7% in medical students, 84.8% in interns and 85.5% in residents. Poor attitude rate was 10.9% in males, 6.3% in females, 4.7% in medical students, 6.8% in interns and 18.8% in residents. Poor practices rate was 90.4% in males, 95.4% in females, 98.4% in medical students, 91.7% in interns and 85.5% in residents.

Poor knowledge rate was 98.2% in students who received any knowledge and information regarding BLS, 93.6% in students who did not receive any formal (certified) training/attended any workshop regarding BLS, and 87% in students who got info about BLS from university courses. Poor attitude rate was 8.7% in students who received any knowledge and information regarding BLS, 11.4% in students who did not receive any formal (certified) training/attended any workshop regarding BLS, and 7.3% in students who got info about BLS from university courses. Poor practices rate was 91.6% in students who received any knowledge and information regarding BLS, 95.5% in students who did not receive any formal (certified) training/attended any workshop regarding BLS, and 92.7% in students who got info about BLS from university courses.

### Correlation between KAP scores

Results of the Pearson correlation test, presented in Table 4, revealed that knowledge was positively correlated with attitude (p-value 0.007, r = 0.147) and practice (p-value < 0.001, r = 0.546). In addition, attitude was positively correlated with practice (p-value 0.008, r = 0.146).

**Table 4:** Results of the Spearman correlation test revealing correlations in between KAP parameters.


		KNOWLEDGE	ATTITUDE	PRACTICES

Knowledge	Pearson Correlation	1	0.147	0.546

	P.value		**0.007**	**<0.001**

	N	330	330	330

Attitude	Pearson Correlation	0.147	1	0.146

	P.value	**0.007**		**0.008**

	N	330	330	330

Practices	Pearson Correlation	0.546	0.146	1

	P.value	**<0.001**	**0.008**	

	N	330	330	330


Statistical correlation was assessed using Pearson correlation test; bold: statistically significant correlation set at 5%.

### Factors affecting KAP of BLS

#### Factors affecting knowledge about BLS

Knowledge was associated with receiving any knowledge and information regarding BLS (p < 0.001), receiving any information from the three sources noting university courses (p = 0.009), hospital workshops/sessions (p = 0.001), and internet courses (p = 0.030). In addition, knowledge was associated with receiving any formal (certified) training/attended any workshop regarding BLS (p = 0.001). Our results showed that students who received any knowledge and information regarding BLS, information from university courses, hospital workshops/sessions, internet courses, and those who received any formal (certified) training/attended any workshop regarding BLS, had higher, but still very limited, knowledge level when compared to others. Correlation Knowledge about BLS in function of demographic characteristics can be seen in Table 5.

**Table 5:** Correlation Knowledge about BLS in function of demographic characteristics.


		N	MEAN	P-VALUE

Gender	Male	156	6.12	0.912

	Female	174	6.15	

Academic Year	4th year medical student	47	4.89	**<0.001**

	5th year medical student	82	5.61	

	6th year medical student	75	6.75	

	7th year medical student	57	6.61	

	1st year of residency	26	6.42	

	2nd year of residency	16	5.63	

	3rd year of residency	12	6.83	

	4th year of residency	9	6.78	

	5th year of residency	6	8.67	

Academic Year	Medical student	129	5.35	**<0.001**

	Interns	132	6.69	

	Resident	69	6.55	

Age		Pearson Correlation coefficient: 0.282 **(p < 0.001)**		


Statistical correlation was assessed using Student t-test and Pearson correlation test; bold: statistically significant correlation set at 5%.

### Factors affecting Attitudes towards BLS

Attitude was not associated with receiving any knowledge and information regarding BLS (p = 0.090), receiving any information from the sources noting university courses (p > 0.05). On the other hand, attitude was associated with receiving any formal (certified) training/attended any workshop regarding BLS (p = 0.007). Our results showed that students who received any formal (certified) training/attended any workshop regarding BLS, had higher knowledge level (mean attitude = 26.3 ± 2.9) when compared to others.

Correlation of attitudes towards BLS depending on demographic characteristics can be seen in Table 6.

**Table 6:** Correlation of attitudes towards BLS depending on demographic characteristics.


		N	MEAN	P-VALUE

Gender	Male	156	25.98	0.897

	Female	174	26.03	

Academic Year	4th year medical student	47	25.91	**0.002**

	5th year medical student	82	26.59	

	6th year medical student	75	25.65	

	7th year medical student	57	26.93	

	1st year of residency	26	24.00	

	2nd year of residency	16	24.50	

	3rd year of residency	12	25.42	

	4th year of residency	9	26.00	

	5th year of residency	6	28.33	

Academic Year	Medical student	129	26.34	**0.018**

	Interns	132	26.20	

	Resident	69	25.00	

Age		Pearson Correlation coefficient: –0.016 **(p = 0.774)**		


Statistical correlation was assessed using Student t-test and Pearson correlation test; bold: statistically significant correlation set at 5%.

### Factors affecting practices of BLS

Practice regarding BLS was associated with receiving any knowledge and information regarding BLS (p < 0.001), receiving any information from the three sources noting university courses (p = 0.002), hospital workshops/sessions (p = 0.001), and internet courses (p = 0.008). In addition, practice regarding BLS was associated with receiving any formal (certified) training/attended any workshop regarding BLS (p = 0.012) Our results showed that students who received any knowledge and information regarding BLS, information from university courses, hospital workshops/sessions, internet courses, and those who received any formal (certified) training/attended any workshop regarding BLS, had higher practice level when compared to others.

Correlation Practices of BLS in function of demographic characteristics can be seen in Table 7.

**Table 7:** Correlation Practices of BLS in function of demographic characteristics.


		N	MEAN	P-VALUE

Gender	Male	156	4.80	0.717

	Female	174	4.73	

Academic Year	4th year medical student	47	3.79	**<0.001**

	5th year medical student	82	4.33	

	6th year medical student	75	5.04	

	7th year medical student	57	5.11	

	1st year of residency	26	5.42	

	2nd year of residency	16	4.44	

	3rd year of residency	12	6.00	

	4th year of residency	9	5.00	

	5th year of residency	6	6.83	

Academic Year	Medical student	129	4.13	**<0.001**

	Interns	132	5.07	

	Resident	69	5.36	

Age		Pearson Correlation coefficient: 0.224 **(p < 0.001)**		


Statistical correlation was assessed using Student t-test and Pearson correlation test; bold: statistically significant correlation set at 5%.

### Predictors of KAP among control group participants

Multiple linear regression was applied to identify predictors of KAP among participants. Knowledge was positively associated with age (p = 0.001), receiving any knowledge and information regarding BLS (p = 0.016), receiving any formal (certified) training/attended any workshop regarding BLS (p = 0.021). Attitude was positively associated with academic year (p = 0.002) and knowledge (p = 0.003). Practice was positively associated with age (p < 0.001) and knowledge (p < 0.001). Results of the linear regression are presented in Table 8.

**Table 8:** Multiple linear regression results showing predictors of KAP among group control participants.


VARIABLES	PREDICTORS	STANDARDIZED COEFFICIENT (β)	P-VALUE	COLLINEARITY	

				TOLERANCE	VIF

Knowledge	(Constant)	0.038	0.980		

	Age	0.215	0.001	0.937	1.067

	Have you received any knowledge and information regarding BLS?	0.845	0.016	0.830	1.204

	Have you received any formal (certified) training/attended any workshop regarding BLS?	0.600	0.021	0.882	1.133

Attitudes	(Constant)	22.010	0.000		

	Year of medical training	1.399	0.002	0.991	1.009

	Knowledge	0.243	0.003	0.991	1.009

Practices	(Constant)	–1.379	0.169		

	Age	0.153	0.000	0.950	1.053

	Knowledge	0.402	0.000	0.950	1.053


## Discussion

### Information and backgrounds of BLS

According to our study, information about BLS was obtained via university courses (53.6%), hospital workshops/sessions (35.8%), internet courses (39.1%), red-cross training (24.8%), pamphlets (15.2%). Knowing that throughout the pandemic, several online training courses took place worldwide to offer BLS knowledge and to avoid communication of misconceptions through non-medical platforms (16). The candidates’ answers to the 13 questions reflect that more than half of the questions (7 questions) were answered correctly by less than 50% of the candidates. These questions were mainly related to the application knowledge of CPR in adults and infants, and their results are not satisfactory. For example, 19.4% knew that they need to conform to foreign body aspiration in case of the sudden expression of choking symptoms, but responsive (case of having food in a canteen). And 17.6% knew that the correct depth of compression in children during CPR is 2 inches, knowing that this is a basic must-known per the CPR guidelines (17).

These results showed a lack of basic knowledge in BLS among almost two-thirds of questioned candidates. This leads to an assumption that either the received training was not enough, or the candidates’ degree of information retention was decreased with time—knowing that this study did not have information regarding the date of receipt of BLS training. In fact, it is suggested that in a training assessment many who attend conventional CPR classes fail to acquire the necessary skills, and the skills that are acquired decline appreciably over the subsequent 6–9 months (18), although this is questionable since, in this case, K and P are poor despite the fact that the concerned persons have received training courses. And lack of information regarding BLS of infants might be related to lack of training or absence of practical application in real life.

Regarding training needs and willingness to attend training on BLS, 58.2% strongly agreed and 35.8% agreed that they would like to undergo BLS training in a workshop/center with hands on practice, under supervision. Knowing that in the previously referred study on training on BLS, only 39 out of 262 participants agreed to pursue the full training curriculum (18).

Furthermore, 75.2% strongly agreed and 20% agreed that BLS training should be a part of your curriculum since full knowledge of BLS isn’t acquired by them.

As for the practical performance of BLS, our study concluded that 39.1% absolutely agreed and 45.2% agreed to perform mouth to mouth ventilation for person of same gender while 30.9% absolutely agreed and 38.2% agreed to perform mouth to mouth ventilation for person of opposite gender. These results correlate with cultural backgrounds and are common as per the literature, where a study including 285 doctors concluded that CPR was preferred over chest compression-only resuscitation (CCR) by 91.6% of the doctors (19). Knowing that when it comes to a confirmed or suspected Covid-19 case, a mouth-to-mouth ventilation is not recommended (20) and this is not related to gender in this case.

Regarding BLS practice, 6 out of 10 questions were answered correctly by less than 50% of the candidates. In fact, a study assessing the training and skills retention of 150 health professionals concluded that only 7.4% of them could answer 75% of the questions accordingly, which suggests a major lack of knowledge in BLS (8). In our study, the best score was for ‘Pulse check should be done via Carotid’ with 90.6% or correct answers and the least score was for ‘The 2010 AHA Guidelines for CPR recommended BLS sequence of steps are chest compressions, Airway, and Breathing with 22.7% correct answers which suggests a lack of specialized orientation towards BLS and CPR. Other questions with correct answers below 50% are related to guidelines of CPR, such as having to switch roles between rescuers.

### KAP scores analysis

Findings showed that medical students had poor knowledge and practice, but good attitudes about BLS. Out of 330 medical students, 90% had poor knowledge level and mean knowledge score was of poor level about 6 ± 2.2 over 13(46.15%) with a minimum of 2 over 13 and a maximum of 12 over 13. Among medical students, 34.2% had good attitudes, 37.3% had excellent attitudes, and mean attitudes score was of good level about 26 ± 3.3 over 30 (86.66%) with a minimum of 10 over 30 and a maximum of 30 over 30. Out of 330 medical students, 93% had poor practice level and mean practice score was of poor level about 4.8 ± 1.8 over 10 (48%) with a minimum of 0 over 10 and a maximum of 10 over 10. These poor knowledge and poor practice scores about BLS are usual according to the literature among medical staff. A similar trial showed that around 70% of questioned candidates did not know rates and depth of chest compression for example (19). A similar study recently done in Egypt showed that 68.3% of the participating junior doctors had insufficient knowledge of CPR, with a score of less than 50% on the questionnaire, 93.8% of medical students, which is a huge percentage, also showed insufficient level of knowledge. But despite this deficit in knowledge, both junior doctors and medical students had positive attitudes towards CPR training (95% in junior doctors and 91%in students) (11). Another study done in Pakistan in 2018 revealed that 36.67% of med students had poor level of knowledge concerning BLS while 31.33% had average knowledge levels and just 1% had excellent level of knowledge when it came to BLS (12). Hence, our results align with literature findings. For example, another study comprising 285 doctors had similar results with a majority of the doctors unaware of the revised rate and depth of chest compressions, CPR was preferred over chest compression-only resuscitation (CCR) by 91.6% of the doctors. Half of the participants rated their knowledge as average. Most stated that they will not be reluctant to perform CPR in an emergency situation. The majority also agreed that BLS training should be an integral part of the medical curriculum (19).

**Figure 2:**
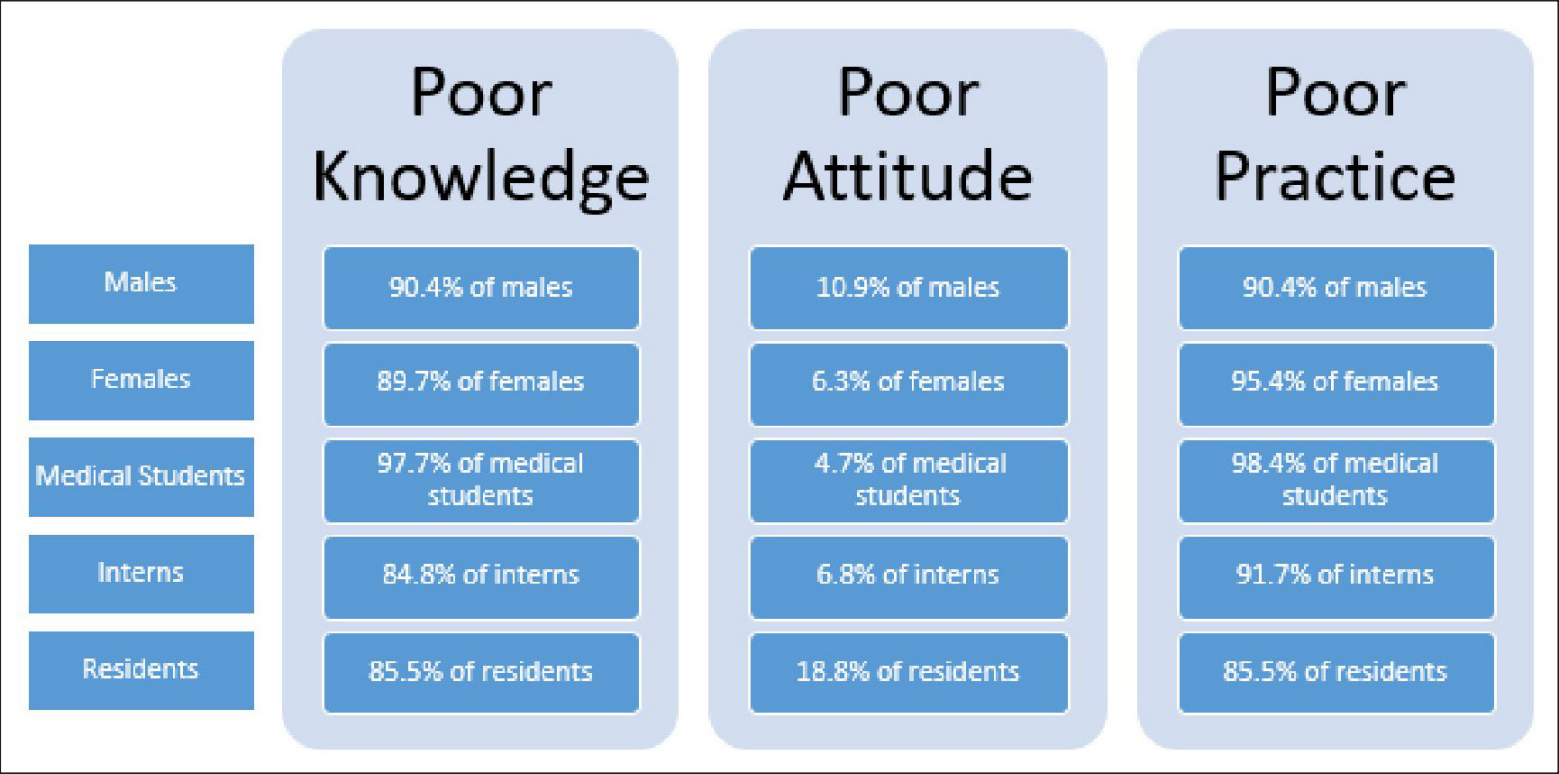
Prevalence of poor knowledge, attitude and practices among males, females, medical students, interns and residents of the LU faculty of medical sciences.

As summarized in Figure 2, most Lebanese University medical students, interns and residents had recorded poor knowledge and practice scores and few of them a poor attitude score about BLS. There is therefore an urgent need to overcome this gap. In general, KAP elements were positively inter-correlated, reflecting direct relationships between knowledge, attitude and practice. In fact, attitude is related to the medical knowledge, however knowledge and practices are poor especially in under graduates because they haven’t faced a real case needing a CPR or haven’t had attended enough courses and trainings.

**Figure 3:**
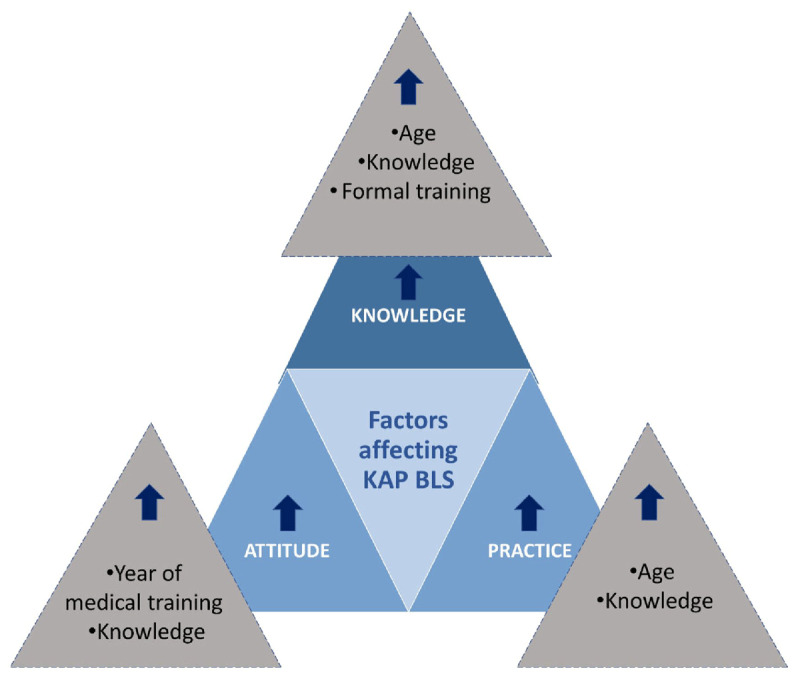
Factors affecting KAP scores about BLS in medical students, interns and residents of the LU faculty of medical sciences.

The study found that Knowledge and Attitude about BLS were not associated with gender but they were associated with age (p < 0.001) and Pearson correlation showed that knowledge about BLS increased with age (r = 0.282) (Figure 3). In addition, knowledge and attitude about BLS were associated with the academic year (p < 0.001) and results showed that knowledge level was higher in fifth year residents, then interns, followed by low grade residents, and medical students. This result underlies that adequate training on BLS is acquired gradually in the academic curriculum and in the hands-on practice inside the hospital. And as per our results, any source of knowledge, either formal or unformal, certified or non-certified, has a direct impact on the knowledge and assimilation of information related to BLS. Our results showed that students who received any formal (certified) training/attended any workshop regarding BLS, had higher attitude level (mean attitude = 26.3 ± 2.9) when compared to others. Similar results were observed regarding Practice assessment (Figure 3).

### Impact of the study

Evidence reflects an alarming gap in the knowledge of medical trainees concerning BLS, especially in developing countries (11,12). Examining the knowledge, practice, and attitude of medical students and junior doctors regarding BLS is of the utmost importance to reduce the morbidity in acute scenarios and promote positive behavior. Appropriate trainings therefore are necessary and allow the promotion of knowledge of BLS and increase the correct use of BLS practices among junior and future physicians. However, such strategies require deep insights into the current state of knowledge, practice, and awareness in order to formulate a targeted approach.

In addition, a completion of a BLS course is not mandatory to graduate from medical school in Lebanese University, which leads to uncertainty about the ability of its students and recent graduates to perform well in an acute life-threatening setting. Hence our study might highlight the importance of integration of efficient BLS training in the undergraduate program of medical schools in Lebanon.

### Study limitations

We did not have a full sample totally showing a proven representation of experience in cardiac arrest of a COVID-19 patient. This is mainly due to the fact that the majority of people engaged in the survey do not work in intensive care units or cardiac care units. Our data lacks information about the date elapsed since our candidates have last received BLS training if any. In addition, any survey filled in a self-reported manner, this study might be subject to a **Reporting Bias;** Consequently, there is a possibility for the participants to refer to external sources to fill the answers related to BLS knowledge and practice, thus affecting the results. We could have overcome this bias by interviewing the participants, but this would be time-consuming. The **central tendency bias** could also be present due to the Likert scale used in evaluating BLS-related attitude. In general, people tend to avoid selecting extreme responses like ‘strongly agree’ or ‘strongly disagree’ in questionnaires and opt for neutral answers. Moreover, this study discusses correlations and strength of association between BLS KAP and studied factors without providing exact causation.

## Conclusion

The study demonstrated that while the attitude is positive, knowledge and practical application remain poor among Lebanese University medical students and junior doctors when it comes to BLS. This lack of proficiency highlights huge potential risks in the daily clinical practice.

In conclusion, the results acquired emphasize the urgent need for a comprehensive reevaluation of BLS training strategies in developing countries. Integrating mandatory practical, continuous hands-on training within the medical curriculum and additionally offering intensive theoretical courses might significantly enhance the preparedness of future medical professionals to handle critical life-threatening situations effectively. Continuous assessment of knowledge, attitude, and practice towards BLS among students and residents would also be beneficial.

## Data Accessibility Statement

Data are available upon request from corresponding author.

## Additional File

The additional file for this article can be found as follows:

10.5334/gh.1368.s1 Supplementary File. Data collection Form.
