# Supplementary material for: Knowledge, Attitude, and Practice of the Lebanese University Medical Students and Junior Doctors on Basic Life Support Practices
Source: Glob Heart. 2024 Nov 13;19(1):86. doi: 10.5334/gh.1368 (PMC11568803; doi:10.5334/gh.1368)
Supplement: Supplementary File. — Data collection Form. [file gh-19-1-1368-s1.pdf]

Data collection Form

1- Demographic and General Data

Please provide us the following demographic data

1.1. Age: _____ Years

1.2. Gender

- Male
- Female

1.3. Year of medical training:

- 4th year medical student
- 5th year medical student
- 6th year medical student
- 7th year medical student
- 1st year of residency
- 2nd year of residency
- 3rd year of residency
- 4th year of residency
- 5th year of residency

1.4. Governorate of residence

- Beirut
- Mount Lebanon
- North Lebanon
- Akkar
- South Lebanon
- Nabatiyeh
- Bekaa
- Baalbeck-Hermel

1.5. Have you received any knowledge and information regarding BLS?

- Yes
- No

1.6. If yes, what were the sources of information? (Multiple answers)

- University courses
- Hospital workshops/sessions
- Internet courses
- Red cross training
- Pamphlets
- Others.

1.7. Have you received any formal (certified) training/attended any workshop regarding BLS?

- Yes (in the last 2 years)
- Yes (over 2 years ago)
- No

2- Knowledge:

2.1. What is the abbreviation of "BLS"?

- Best Life Support
- Basic Life Support
- Basic Lung Support
- Basic Life Services

2.2. When you find someone unresponsive in the middle of the road, what will be your first response? (Note: You are alone there)

- Open airway
- Start chest compression
- Look for safety
- Give two breathings

2.3. If you confirm somebody is not responding to you even after shaking and shouting at him, what will be your immediate action?

- Start CPR
- Activate EMS
- Put him in recovery position
- Observe

2.4. What is the location for chest compression?

- Left side of the chest
- Right side of the chest
- Center of the chest on lower half of breast bone
- Xiphisternum

2.5. What is the location for chest compression in infants?

- One finger breadth below the nipple line
- At the intermammary line
- One finger breadth above the nipple line
- At xiphisternum

2.6. How do you give rescue breathing in infants?

- Mouth-to-mouth with nose pinched
- Mouth-to-mouth and nose
- Mouth-to-nose only
- Mouth-to-mouth without nose pinched

2.7. Depth of compression in adults during CPR

- At least 2 inches
- 2 1/2 – 3 inches
- 1 – 1 1/2 inches
- 1 1/2 inch

2.8. Depth of compression in children during CPR

- 2 inches
- 2 – 2 1/2 inches
- 1 – 1 1/2 inches
- 1/2 – 1 inch

2.9. Depth of compression in neonates during CPR

- 1 1/2 – 2 inches
- 2 – 2 1/2 inches
- 1 inch
- Approximately 1 1/2 inch

2.10. Rate of chest compression in adult and children during CPR

- At least 100 / min
- Approximately 100 / min
- 80 / min
- 120 / min

2.11. What does abbreviation AED stands for?

- Automated External Defibrillator
- Automated Electrical Defibrillator
- Advanced Electrical Defibrillator
- Advanced External Defibrillator

2.12. What does abbreviation EMS stands for?

- Effective Medical Services
- Emergency Management Services
- Emergency Medical Services
- External Medical Support

2.13. If you and your friend are having food in a canteen and suddenly your friend starts expressing symptoms of choking <u>but responsive</u>, what will be your first response?

- Give abdominal thrusts
- Give chest compression
- Confirm foreign body aspiration by talking to him
- Give back blows

3- Attitude

3.1. Do you think BLS is necessary?

- Yes
- No
- Can't say or not sure

3.2. If yes, how necessary it is?

- Very much important
- Important
- Indifferent

3.3. Have you ever voluntarily performed BLS?

- Yes
- No
- Performed but not voluntarily

3.4. Would you perform mouth to mouth ventilation for person of same gender?

- Yes
- No
- Hesitant

3.5. Would you perform mouth to mouth ventilation for person of opposite gender?

- Yes
- No
- Hesitant

3.6. Would you like to undergo BLS training in a workshop/centre with hands on practice under supervision?

- Yes
- No
- Not sure

3.7. Do you think that BLS training should be a part of your curriculum?

- Yes
- No
- Not sure

4- Practice

4.1. The 5 links in the adult Chain of Survival include all of the following EXCEPT:

- Early CPR
- Integrated post cardiac arrest care
- Advanced airway placement
- Rapid defibrillation

4.2. How often should rescuers switch roles when performing 2-rescuer CPR?

- After each cycle
- After 2 cycles
- After 5 cycles
- After 10 cycles

4.3. The initial Basic Life Support (BLS) steps for adults are:

- Assess the victim, give 2 rescue breaths, defibrillate, start CPR
- Assess the victim, activate EMS and get AED, check pulse, start CPR
- Check pulse, give rescue breaths, assess the victim, defibrillate
- Assess the victim, start CPR, give 2 rescue breaths, defibrillate

4.4. Where should you attempt to perform a pulse check in adult?

- Carotid
- Brachial
- Ulnar
- Temporal

4.5. The compression to ventilation ratio for the lone rescuer giving CPR to victims of ANY age is:

- 15:1
- 15:2
- 30:1
- 30:2

4.6. The proper steps for operating an AED are:

- On the AED, attach electrode pads, shock the patient, analyze the rhythm
- On the AED, attach electrode pads, analyze the rhythm, clear the patient, deliver shock
- Attach electrode pads, check pulse, shock patient, analyze rhythm
- Check pulse, attach electrode pads, analyze rhythm, shock patient

4.7. The 2010 AHA Guidelines for CPR recommended BLS sequence of steps are:

- Chest compressions, Airway, Breathing
- Airway, Breathing, Check Pulse
- Airway, Breathing, Chest compressions
- Chest compression, Airway placement, Breathing

4.8. Signs of severe airway obstruction include all of the following EXCEPT:

- Poor air exchange
- High-pitched noise while inhaling
- Unable to cry
- May wheeze between coughs

4.9. In an adult with an advanced airway in place during 2-rescuer CPR, breaths should be administered how often?

- Every 5 seconds
- Every 5-6 seconds
- Every 6-8 seconds
- Every 10-12 seconds

4.10. The critical characteristics of high-quality CPR include which of the following?

- Starting chest compressions within 10 seconds of recognition of cardiac arrest
- Push hard, push fast
- Minimize interruptions
- All of the above

Answers:

2.1. B

2.2. C

2.3. B

2.4. C

2.5. A

2.6. B

2.7. A

2.8. A

2.9. D

2.10. A

2.11. A

2.12. C

2.13. C

4.1. C

4.2. C

4.3. B

4.4. A

4.5. D

4.6. B

4.7. A

4.8. D

4.9. C

4.10 D
